# Compound dataset and custom code for deep generative multi-target compound design

**DOI:** 10.2144/fsoa-2021-0033

**Published:** 2021-04-30

**Authors:** Thomas Blaschke, Jürgen Bajorath

**Affiliations:** 1 Department of Life Science Informatics & Data Science, B-IT, LIMES Program Unit Chemical Biology & Medicinal Chemistry, Rheinische Friedrich-Wilhelms-Universität, Friedrich-Hirzebruch-Allee 6, Bonn, D-53113, Germany

**Keywords:** biological assays, computer-aided drug design, generative modeling, large-scale data analysis, machine learning, multi-target compounds

## Abstract

**Aim:**

Generating a data and software infrastructure for evaluating multi-target compound (MT-CPD) design via deep generative modeling.

**Methodology:**

The REINVENT 2.0 approach for generative modeling was extended for MT-CPD design and a large benchmark data set was curated.

**Exemplary results & data:**

Proof-of-concept for deep generative MT-CPD design was established. Custom code and the benchmark set comprising 2809 MT-CPDs, 61,928 single-target and 295,395 inactive compounds from biological screens are made freely available.

**Limitations & next steps:**

MT-CPD design via deep learning is still at its conceptual stages. It will be required to demonstrate experimental impact. The data and software we provide enable further investigation of MT-CPD design and generation of candidate molecules for experimental programs.

## Lay abstract

Small molecules with well-defined activity against multiple biological targets are increasingly considered for therapy of complex diseases. Generating such compounds is far from being trivial. Therefore, deep machine learning, a form of artificial intelligence, is applied to aid in this process. For this purpose, we have generated a data set and software that we make freely available to further advance deep learning for designing multi-target compounds.

## Graphical abstract

A group of three compounds with multi- or single-target activity or no activity (no target). For grouping of compounds according to a set of targets, confirmed activity or inactivity against all targets is taken into account.

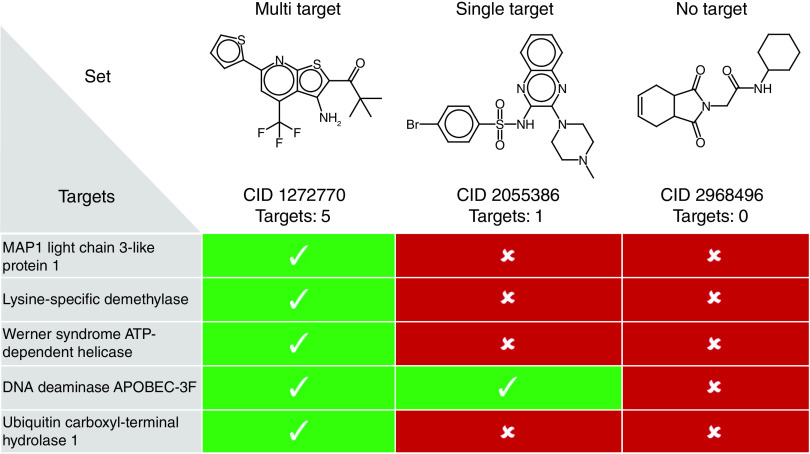

In pharmaceutical research, multi-target compounds (MT-CPDs) are increasingly considered for the treatment of complex pathologies such as cancer or neurodegenerative diseases, due to their ability to elicit polypharmacological effects [1–3]. Generating compounds that are capable of selectively interacting with two or more targets is challenging [3] and benefits from computational support [3,4]. For example, computational concepts such as pharmacophore modeling are applicable to aid in creating candidate MT-CPDs [4]. In drug design, machine learning using deep neural network architectures receives increasing attention [5]. One of the areas in which deep learning has become a focal point is *de novo* compound design via generative modeling [5,6]. This approach aims at generating populations of novel compounds with desired properties such as a specific biological activity. For this purpose, different types of deep neural network architectures and learning strategies have been adapted [6]. While increasing numbers of applications are reported, deep generative modeling has thus far not been applied to MT-CPD design. As summarized in the Methodology section, we have recently carried out a proof-of-concept investigation to specifically evaluate this approach for MT-CPD design (unpublished). To enable this study, the generation of custom code and a specialized molecular test system was required. In this Data Note, we describe the test system and code and report an open access deposition making the data and code freely available to the scientific community. We hope that the availability of these computational tools will motivate additional applications of generative modeling for MT-CPD design, help to further evaluate the approach, and demonstrate its relevance for drug discovery.

## Methodology

### Analysis concept

To evaluate the potential of generative modeling for MT-CPD design we attempted to fine-tune a general-purpose model, as further described below. For this study, we required a suitable compound test system and custom code for fine-tuning. While the results of our proof-of-concept investigation are yet to be reported, making the test system and custom code publicly available will enable other investigators to explore MT-CPD design, as further detailed below.

### Benchmark system

We aimed to focus the analysis on compounds with experimentally confirmed activity or inactivity against biological targets. Therefore, screening compounds were systematically extracted from PubChem [7] applying the following data confidence and selection criteria:

- Assays for individual human targets were selected;
- Qualitative activity annotations were considered: ‘active’ or ‘inactive’;
- Inconsistently annotated or revoked assays were disregarded as well as assays imported from other databases (for external assays, negative test results were mostly missing);
- Assays with an unusually high hit rate >2% were eliminated;
- Screening compounds with aggregation or other assay interference (artifact) potential [8–10] were discarded;
- Three categories of compounds were assembled:
  – MT-CPDs: compounds with activity against five or more targets;
  – Single-target compounds (ST-CPDs): compounds with activity against only one target and confirmed inactivity against at least four other targets;
  – Inactive or ‘no-target’ compounds (NT-CPDs): compounds with no reported activity, but confirmed inactivity against at least five targets.

Application of these criteria ensured that test compounds were classified taking negative assay results (inactivity) into account and MT-CPDs were clearly distinguished from ST- and NT-CPDs based on the number of experimental target annotations. Criteria 4 and 5 were applied because the assignment of MT-CPDs is highly vulnerable to potential experimental artifacts (false positives).

On the basis of our selection criteria, a total of 2809 MT-, 61,928 ST- and 295,395 NT-CPDs were obtained.

### MT-CPD modeling

For deep generative design, we adapted the REINVENT 2.0 model [11] that is publicly available. This model was originally trained on SMILES representations [12] of approximately 1.4 million bioactive compounds from ChEMBL [13] to learn the SMILES syntax and generate valid strings representing new compounds. We note that the majority of compounds from ChEMBL are ST-CPDs [14]. Details of the model are reported in the original publication [11].

MT-CPD design was attempted by further extending the REINVENT 2.0 model for fine-tuning on MT-CPDs through transfer learning [15]. Following this idea, a general-purpose model capable of generating valid SMILES strings is subjected to a second training phase (fine-tuning) using a confined set of compounds with desired properties (e.g., MT activity). The goal of the second training phase is learning characteristic features of these compounds and preferentially generating others with corresponding features. Fine-tuning via transfer learning was facilitated with in-house custom code further described below.

For fine-tuning, 1000 randomly selected MT-CPDs were used as a training set. The remaining 1809 MT- and 61,928 ST-CPDs as well as an equally sized random subset of NT-CPDs were used as test sets.

## Exemplary results

### Experimental compound evaluation

**Figure 1.**
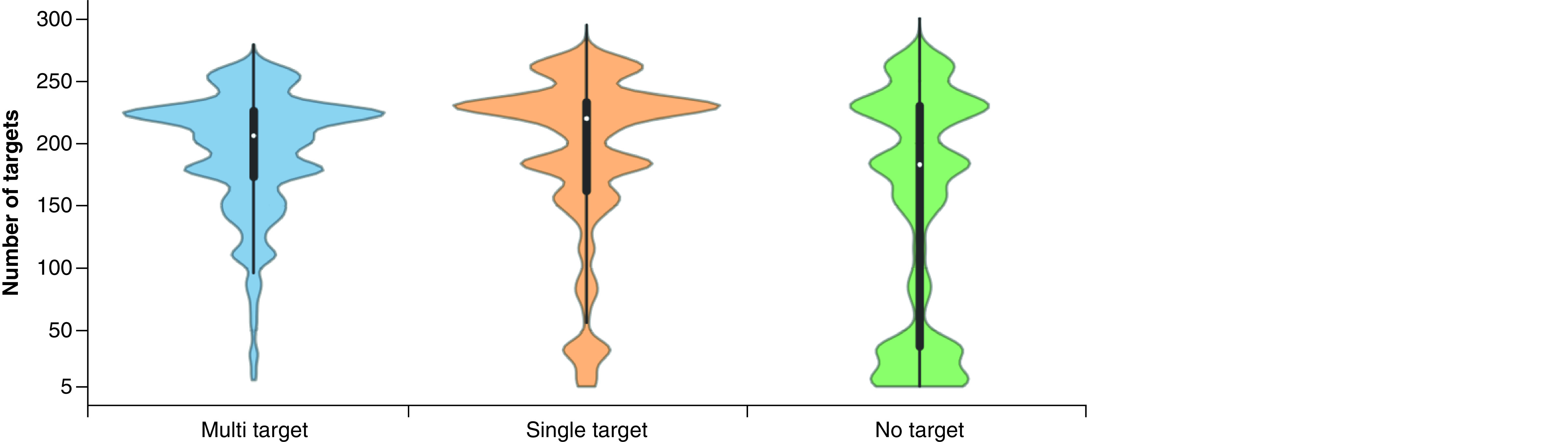
Target frequencies. Violin plots report the distributions of targets against which MT-, ST- and NT-CPDs were experimentally tested. A violin plot combines a boxplot (black bar in the center) with a density plot (colored shape). In the density plot, the distribution shape of the data is visualized; the width of the density plot is proportional to the value frequency. In the boxplot, the LAV (bottom end of black line), lower quartile (lower boundary of the central bar), median (white dot in the bar), upper quartile (upper boundary of the bar) and UAV (top end of black line) of the value distribution are reported. LAV: Lower adjacent value; MT-CPD: Multi-target compound; NT-CPD: No-target compound; ST-CPD: Single-target compound; UAV: Upper adjacent value.

Figure 1 shows the distributions of the number of targets against which newly assembled MT-, ST- and NT-CPDs were tested. The distributions reveal that these compounds were overall extensively assayed (with median values of 206, 220 and 183 targets per compound, respectively), hence lending credence to their classification as MT-, ST- and NT-CPDs.

### Fine-tuning & model assessment

The REINVENT model was fine-tuned over 200 epochs. Over the course of fine-tuning, we monitored whether the model learned features distinguishing between MT-CPDs and ST-/NT-CPDs and preferentially generated MT-CPDs. Fine-tuning was guided by minimizing the negative log-likelihood (NLL) of the SMILES strings of MT-CPDs from the training set. NLL values quantitatively estimate the likelihood that the model will regenerate a known compound at each stage of the fine-tuning process. After each epoch one million SMILES strings were sampled and canonicalized. On the basis of canonical SMILES, regenerated test set compounds were identified, and their NLL calculated.

### Proof of concept

**Figure 2.**
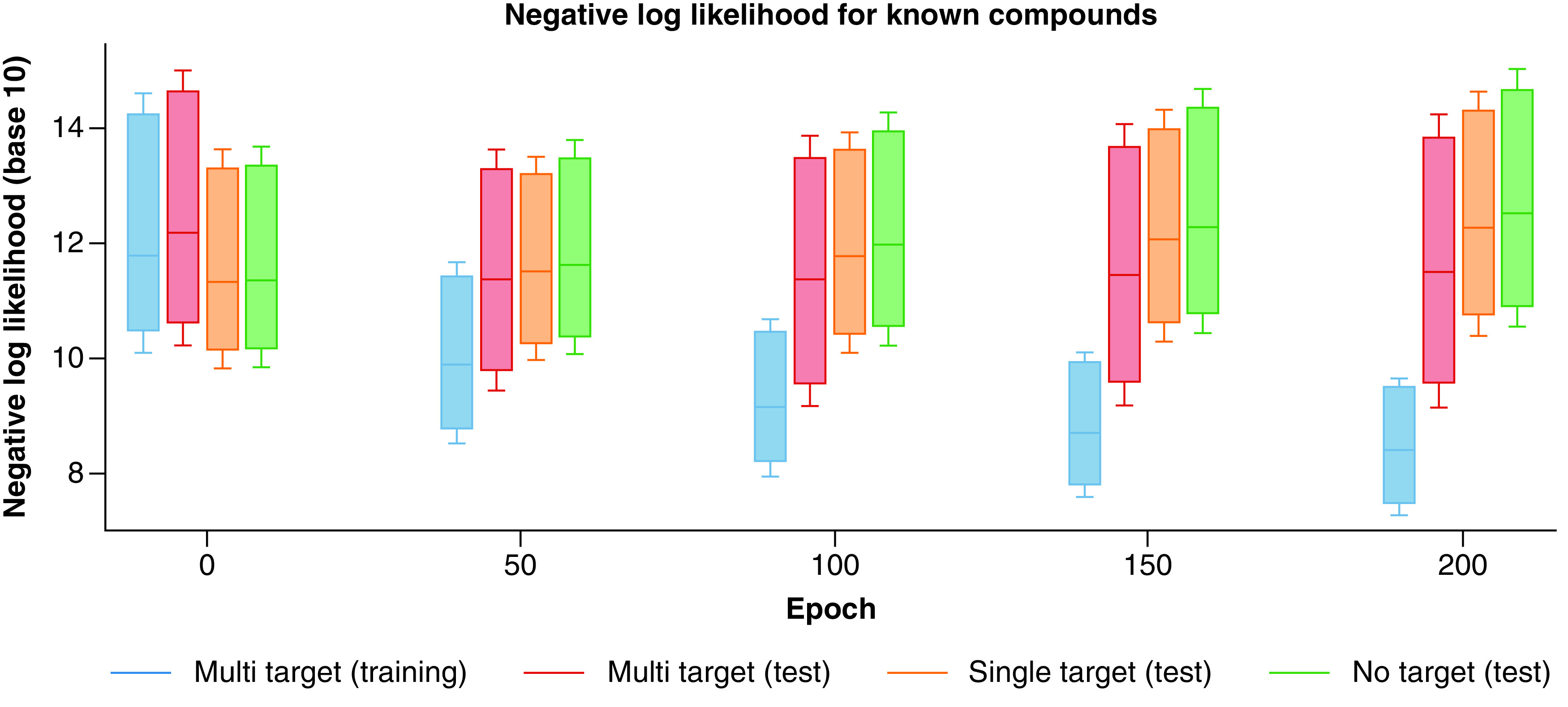
Model evaluation. Boxplots report the distribution of NLL values for MT-, ST- and NT-CPDs during fine-tuning of the REINVENT model after 0, 50, 100, 150 and 200 epochs. For clarity, statistical outliers are omitted. MT-CPD: Multi-target compound; NLL: Negative log-likelihood; NT-CPD: No-target compound; ST-CPD: Single-target compound.

Figure 2 monitors the distribution of NLL values across the fine-tuning process. At the starting point (epoch 0), the REINVENT model was approximately four times more likely to generate ST- or NT-CPDs than MT-CPDs. Initially, approximately 3% of compounds of test compounds from all three sets were regenerated. As shown in Figure 2, the likelihood of generating MT-CPDs systematically increased during fine-tuning, whereas the likelihood of producing ST-/NT-CPDs decreased. After 200 epochs, approximately 85% and 21% of training and test MT-CPDs were regenerated, in contrast to only 3–4% of test ST-/NT-CPDs. At this stage, the fine-tuned model was approximately six times more likely to generate an MT- than ST-CPD. The trend to preferentially produce MT-CPDs was already detectable after 50 epochs.

Taken together, the findings provided proof-of-principle for the ability of generative modeling to yield MT-CPDs via transfer learning (full results will be reported elsewhere). Since modeling was exclusively based on SMILES representations, an important condition for successful generation of MT-CPDs was the presence of structural patterns distinguishing MT- from ST-/NT-CPDs, which the model could detect, learn and exploit. Hence, the results also provide further evidence that such patterns exist. After fine-tuning, the model generated on average 22 and five structural analogs of per regenerated training and test MT-CPD, respectively. Thus, the model recognized structural characteristics and utilized them for compound design, also leading to the generation of analogs.

Using our compound test system and custom code, MT-CPD design can be investigated in different ways using the further extended public domain REINVENT 2.0 framework.

## Data

### Compounds

All of 2809 MT-, 61,928 ST- and 295,395 NT-CPDs are provided as three subsets in a tab-delimited text file (.tsv format). For each compound, the canonical SMILES representations, class label and PubChem ID are given. In addition, for each compound, the following is reported:

- List of PubChem assays (IDs) in which it was tested;
- Number of positive assay results;
- Corresponding UniProt IDs for the PubChem assay targets;
- Total number of targets the compound was active against.

### Custom code

The provided source code contains the routines used for:

- creating the data sets;
- fine-tuning of the REINVENT model;
- analyzing newly generated compounds;
  In addition, the code contains modifications to REINVENT 2.0 to:
- ensure reproducibility of fine-tuning;
- facilitate compound sampling.
- calculate NLL values for test compounds.

Further documentation is provided in the deposition.

### Data deposition

Compound sets and source code are made freely available in an open access deposition on GitHub [16].

## Limitations & next steps

Different deep generative modeling approaches are currently applied to various compound design tasks, often with high expectations. While scientifically stimulating, true impact of generative modeling on experimental programs and unprecedented advances are yet to be demonstrated on a larger scale. This also applies to MT-CPD design, which currently is still at its conceptual stages. To further evaluate and advance these deep learning approaches, we consider scientific rigor, reproducibility and data sharing to be essential. Accordingly, we hope that the availability of our data and code will motivate additional investigations of MT-CPD design. For the compound set we provide, general accuracy limitations associated with assay readouts apply. However, the data curation protocol balanced potential inaccuracies and took advantage of negative test results, which was a major motivation for focusing on screening data. As the next steps, we intend to investigate alternative learning strategies for *de novo* MT-CPD generation and compare the outcome to knowledge-based design approaches.

**Executive summary**

- Relevance of MT-CPDs is discussed;
- Deep generative modeling is introduced.

**Methodology**

- The screening data curation protocol is detailed;
- Model fine-tuning for MT-CPD design via transfer learning is introduced.

**Exemplary results**

- Compound assay statistics are reported;
- Proof-of-concept for generative MT-CPD design is provided.

**Data**

- Compound data and source code are described;
- The data and code deposition is specified.

**Limitations & next steps**

- The need to assess measurable impact of generative modeling is discussed;
- Studies to further evaluate and improve the approach are outlined.
